# Facebook Enables Disaster Research Studies: The Use of Social Media to Recruit Participants in a Post-Disaster Setting

**DOI:** 10.1371/currents.dis.f4a444e1f182776bdf567893761f86b8

**Published:** 2017-01-19

**Authors:** Karin Hugelius, Annsofie Adolfsson, Mervyn Gifford, Per Örtenwall

**Affiliations:** School of Health Sciences, Örebro University, Örebro Sweden and Karlskoga Hospital, Orebro County Council, Karlskoga, Sweden; School of Health Sciences, Örebro University, Örebro, Sweden; School of Health Sciences, Örebro University, Örebro, Sweden; Sahlgrenska Akademin, Gothenburg University, Gothenburg, Sweden

## Abstract

Introduction: Disaster research entails several methodological challenges, given the context of a disaster. This article aims to describe and evaluate the use of Facebook as a tool to recruit participants for a self-selected Internet sample using a web-based survey in a post-disaster setting in the Philippines after the Haiyan typhoon hit parts of the country in November 2013.

Method: An invitation to a web-based survey about health was posted on several Facebook pages during a ten-day period.

Results: In total, 443 individuals who had survived the Haiyan typhoon participated in the study. The demographics of the study sample were similar to the general demographics in the Philippines, considering gender, age distribution and level of education.

Discussion: The study showed that the use of social media to recruit participants for disaster research could limit several of the practical and ethical challenges connected to disaster research. However, the method demands access to the Internet and requires several strategic considerations, particularly concerning non-probability sample biases and generalization as well as an active approach from the researcher.

## Introduction

Evaluating the effects of a disaster intervention entails several methodological challenges, given the context of a disaster which usually occurs with no or short notice and causes severe damage to infrastructure^1^^,^ 2 ^,^ 3. There is still no consensus on methodologies for reporting and evaluating effectiveness and benefits of humanitarian interventions for the individuals or societies affected by a disaster. Remarkably few disaster health interventions are evidence-based^3^^,^ 4 ^,^ 5. One major challenge is the speed that is required to capture critical episodes in the first phases of a disaster^3^. There is typically no time to train research teams, for example, or to develop instruments^3^. The nature of sudden-onset disasters may also complicate normal formal procedures such as financing and ethical approval procedures that normally need to be planned long before the research is actually performed^6^. Practical challenges include access to the research area, particularly in the early stages after the disaster when logistical problems can make it impossible to physically access and remain in the area^6^. Security concerns, both for the researcher and for the study participants, must be carefully analysed^6^. Recruitment procedures and participation for both qualitative data studies and quantitative studies may be complicated by the fact that potential participants can be temporarily displaced, injured, or severely traumatized and, therefore making impossible to access participants for research purposes^1^. Lack of baseline data is a common problem when trying to evaluate the effects of disaster interventions or value the findings^3^.

Several ethical considerations must also be made in the research process. In the early stages after a disaster, the gap between resources and needs is acute. At the same time, every person on the ground, including rescue workers, constitutes a burden for the affected community and the disaster response system, because they require resources such as lodging, food and water, transportation etc. The idea of conducting research immediately after a disaster may therefore raise ethical considerations regarding the role and benefits of the presence of researchers in the disaster area^2^. Including disaster-affected people in research requires a sensible approach to ensure that no further harm is caused by the research^1^. However, research is generally well tolerated by distressed people^2^, and failing to conduct disaster-related research because of ethical or methodological challenges may also be unethical^2^.

Several strategies have been described to overcome some of the methodological challenges mentioned above. Field studies which to a greater or lesser extent are integrated in disaster response interventions are often reported as “lessons learned”^1^. Cooperating with local investigators or partners and including non-governmental organizations or relief teams can be a way to collect data or access the area^1^^,^ 7. Disaster exercises used for training purposes offer another way to study the development and evaluation of disaster preparedness and disaster interventions and are assumed to serve as more easily accessible research areas than actual disasters^8^^,^ 9. However, other challenges remain to be addressed, including challenges of timing, recruitment of participants, and the need for some type of tracking mechanism to conduct traditional case-control, matched samples, or follow-up studies^3^.

Currently, almost 40% of the population around the world has access to the Internet. Social media has been used to communicate with communities, authorities, relief organizations, and individuals before, during, and after disasters^10^. The use of social media for the recruitment of participants in health-related research has provend to be an effective way of reaching study populations, as well as being cost-effective and providing an opportunity for follow-up research^11^. Although this recruitment method is associated with sample bias and selection bias, it offers the possibility of reachinf a wide field of study participants^12^. If social media could be used in disaster research, several of the methodological challenges of such research would be reduced. The Philippines, for example, which is one of the most disaster-prone countries in the world, had approximately 34 million registered Facebook accounts in 2014^13^. In the general Philippine population, 81% of women and 76% of men own a cellular phone, and approximately 50% use social media for networking^14^.

In November 2013, the Haiyan super typhoon, locally called Yolanda, hit parts of the Philippines, affecting approximately 14 million people. The typhoon caused severe damage to infrastructure, including not only electricity, water, sanitation, and telephone lines but also official services such as medical facilities 15. Information about the availability of Internet facilities for the public does vary, but most likely, Internet was not available for the first week and there was reduced availability for at least two weeks 16. One of the most affected areas was the regional capital in Leyte province, Tacloban, which normally has approximately 250 000 residents, but at the time of the disaster had approximately 500 000 persons in the area. A research project was conducted to evaluate the health effects of the Haiyan typhoon and the effects of disaster radio as a form of disaster intervention. In one study aiming to discuss the use of a web-based survey for future evaluation of health effects from disaster response interventions and to describe the health of survivors’ and health professional´s 30 months after a natural disaster, social media was used to recruit participants.

The aim of this paper was to describe and discuss the use of Facebook as a tool to recruit participants for a self- selected Internet sample for a web- based survey in a post-disaster setting in the Philippines.

## Methods

For this evaluation, the recruitment and data collection procedure was manually and automatically monitored by the survey system used and by the first author (KH). Data from official sources^14^^,^ 17 were used to compare the study sample and the general population.

*Recruitment and data collection procedure:* Recruitment and data collection procedure: The web-based survey, which aimed to describe health in a post-disaster setting, was created and posted as an Internet link using the www.abcde.xxx format. The study was introduced on Facebook as a short study invitation followed by the link itself. Individuals who clicked the link were given further study information and presented with an informed consent declaration, which had to be accepted before the actual survey could be accessed. The survey was anonymous, and no personal data such as name, Facebook account name, e-mail address, or other personal or trackable data were requested or saved. The short invitation text was first formulated as “Invitation to a research study on health after a natural disaster”. After five days, the invitation was changed to a shorter and more proactive text: “Did you experience the Yolanda typhoon?“ The invitation was posted on five different closed or public Facebook group pages. One group invited Haiyan disaster survivors to share their disaster stories, two groups were aimed at the general population in the specific geographic area of the study, and the remaining two groups were aimed at specific professional and/or voluntary emergency responders (see Table 1). In total, the group pages had approximately 43,250 members, but no information could be obtained regarding how many of these members were actively using Facebook or about whether any individuals were members of more than one of the groups included in this study. To post the study invitation on the closed (non-public) Facebook pages, the principal researcher contacted the group administrator and asked to be invited. The invitation was also spread through individuals sharing the link in their own Facebook networks. The data collection continued for ten days, starting at 10 pm (Philippine local time) on the first day and closing at 7 am (local time) on day 10 (see Table 1). To attract more participants, the invitation message was re-posted in several groups when it had shifted down in the message “flow” (see Figure 1). No compensation or reward was given to the participants for participation in the study.

*Ethical considerations:* The study was done in accordance with the World Medical Association Declaration of Helsinki approved by the VIII Regional Ethical Committee of Eastern Visayas, The Philippine, document number EVHRDC-ERC 2015-05.

## Results

*Study sample and demographic data*: In total, 443 participants answered the survey; 40% were male (n= 175), and were 60% female (n= 267). The majority, 59% (n= 263), were aged between 26 and 65 years old; 37% (n=162) were aged between 18 and 25, and 4% (n=65) were older than 66 years. Of the participants, 3% (n= 11) had elementary school as their highest completed level of education, 43% (n=190) had completed high school, and 53% (n= 235) had completed university studies. Approximately 1% (n=4) answered “other level of education”; see Table 1. Data from official sources were used to describe the characteristics of the study sample in relation to the general population (see Table 2). The study sample had a slightly higher female representation than the general population. Because of different ways of presenting age, an exact comparison was not possible, but the age distribution was generally similar in the study sample to that in the general population.

*Participation rate and researcher activity:* The participation rate was monitored several times a day during the data collection. The number of participants per day varied from 14 to 101, with a median of 37 participants per day (see Table 2 and Figure 1). The headline of the invitation was changed on day five to a shorter one that was possible to read without actively clicking on the Facebook statement. This new headline was used thereafter. The first author (KH), who manually monitored the response rate, had the impression that the response rate increased if she was online with her own Facebook page open. A few (n=3) potential study participants contacted KH through personal Facebook messages to confirm that the study invitation was not a fake, spam, or a sales attempt. No information was obtained about how many people had accessed the invitation but had chosen not to participate in the study.

*Ways of being invited and means of answering the survey*: Most participants had been invited by noticing the invitation on a general Facebook page, such as a private or public Facebook group page: 70% (n= 309). Some 24% (n= 106) had been invited through a personal Facebook invitation, such as from a friend or colleague. A total of 4% (n=18) had received the invitation from a friend or colleague without using Facebook. An absolute majority answered the survey using a mobile phone 77% (n= 339), followed by those who used a computer (18%; n= 80) or tablet (3%; n= 12).

**Table 1. Characteristics in the general population and study sample:** 

Factor	General population	Study sample
Gender*	50% male	39% male
	50% female	61% female
Age in percentage of the total population**		
15-24 years	37%	18-25 years; 37%
25-54 years	37%	26-65 years; 59%
54-65 years	6%	
≥65 years	4%	4%
Highest educational level***		
No education	7%	0%
Elementary school	11%	3%
High school	17%	43%
University	48%	53%
Urban population	49%	76%
* Philippine Statistics Authority** In the Eastern Visayas region*** Philippine National Demographics and Health Survey 2013		

**Table 2. Recruitment actions and number of participants:** 

Day	Weekday	Invitation posted on Facebook***	Actions taken by the researcher	Number of new participants (per day)	Total number of participants
1	Saturday*	Posted in group 1 and on two individual pages		19	19
2	Sunday	Posted in group 2		15	34
3	Monday			31	65
4	Tuesday	Posted in group 3		16	81
5	Wednesday	Re-posted in group 1	New headline for the invitation in group 1,2, 3	57	138
6	Thursday			101	239
7	Friday			81	320
8	Saturday	Posted in group 4 and 5		65	385
9	Sunday	Re-posted in group 1		44	429
10	Monday**			14	443
* Survey opened at 10pm local time** Survey closed at 7am local time*** Group 1: Open Facebook group where Haiyan survivors could share their disaster experiences (40 000 members)Group 2: Closed Facebook group for nurses in the actual region (20 members)Group 3: Closed Facebook group for disaster volunteers in the actual region (80 members)Group 4: Open Facebook group for the general population within the region, open for all types of questions (3000 members) Group 5: Closed Facebook group for the general population in the region (150 members)					

**Number of participants per day and in total:**
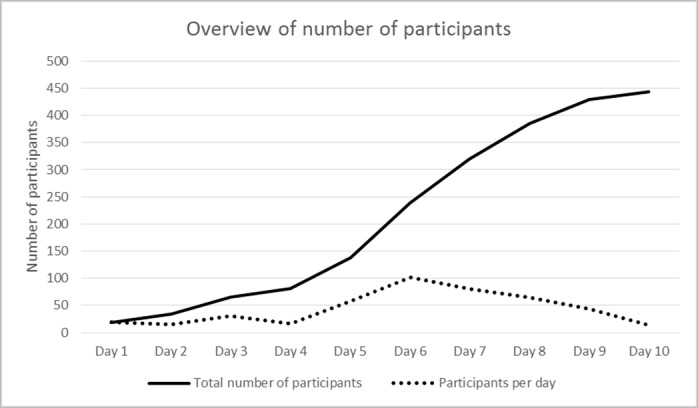

## Discussion

This study shows that Facebook was useful for recruiting a self-selected Internet sample for a web-based survey in a post-disaster setting and reduced a number of practical challenges related to post-disaster research.

There are several advantages of using social media to recruit participants for disaster research. First, the method makes it possible to recruit participants without having the researcher physically located in the area of study, which eliminates several practical, security, and ethical considerations. Second, the use of social media facilitates follow-up studies. In disasters, it is common for people to be forced to relocate from their ordinary homes to evacuation centers or other temporary locations^19^. Ordinary postal delivery services and telephone lines would probably be hampered for quite some time, which would complicate follow-ups based on these traditional methods. In addition, one well-known source of selection bias in disaster evaluations is that these evaluations normally only include participants who have remained in the area, and ignore those who have temporarily or permanently left^3^. Once an individual has agreed to participate and share their contact information, social media allows participant follow-up regardless of their physical location. Third, this study shows that the method can provide sample population that includes participants of different ages, genders, and educational levels.

This study sample had a slightly higher number of female participants. In disaster relief work, as well as in disaster research, women are sometimes mentioned as a specific group that has been marginalized^20^, and so this possible skewness could be seen as a benefit rather than a problem. It is possible that Facebook users in general have a higher educational degree, or are more used to using social media, and this could have influenced the sample characteristics. It is also likely that the type and number of Facebook groups where the study invitation is posted will influence the sample, which must be considered. In terms of the highest level of education, the study sample and general population did not differ, except for those who reported their highest level of education was elementary school, which could be explained by the fact that this study invited only participants over 18 years of age.

Facebook is only one of many social media sources. It may differ as to which social media is being used for targeting general or specific populations in the world and among, for example, age groups in some populations. This must be considered when choosing what type of social media should be used for recruiting study participants. In future comparisons between self-selected Internet samples and the general population, statistical analyses are suggested.

The use of Facebook in self-selected Internet sample post-disaster settings also has several disadvantages. Obviously, Internet access as well as power are needed, which may be a problem in both urban and non-urban areas after a disaster, particularly in the early stage after a disaster. The new joint strategy on communication in disasters^21^ notes that providing the affected community with Internet access will be more strongly prioritized in any future response strategy. Hopefully, this will not only contribute to enabling social contacts and information sharing for those affected but will also contribute to research opportunities. As in all research, the sample strategy must fit into the overall design. In a review of disaster health studies conducted in natural disaster settings, the sample size varied from eleven to over 5,000 participants, with a median of 150 participants^22^. The self- selected Internet sampling used in this study can be said to be a non-probability, convenient sample. Only 1% of the target population participated in the study. To what extent findings from such a sample can generalized to the general population or to disasters in general can be discussed.

However, conducting traditional randomized trials and using traditional sampling procedures in post-disaster settings is a real challenge, given the methodological and practical possibilities^1^^,^ 23. The use of Facebook to recruit participants will not solve this fundamental problem with selection biases but can still make an important contribution to disaster research. Although Facebook may sound like an easy way to recruit participants, experience from this study shows that the successful use of the method demands activity from the researcher. We recommend that the recruitment procedure and progress should be monitored on a daily basis to adjust posting procedures and correct any inappropriate use of the study invitation. The main researcher (KH) had the impression that with the online presence of the researcher, more people chose to participate in the study. One of the challenges of using social media might be to convince potential participants that the invitation and survey is not a spam or virus-infected link. The presence of a personal contact person may reduce those suspicions.

In this study, the invitation text was modified after a few days. We recommend using a very short and even somewhat non-traditional phrasing for the invitation. Social media plays an important role in disaster assessment, disaster response, risk communication, and research into crisis communication^10^^,^ 13. It also provides a forum for survivors to express emotions and tell stories about the disaster^10^. During this study, several groups and forums specifically used by Haiyan (Yolanda) survivors were identified. These forums may be of interest not only to the affected population but also to researchers wishing to further understand the processes and needs of the affected population. As some of the participants in this study expressed, the use of Facebook to advertise disaster research sent the message to the affected community that someone cared, and that the disaster survivors could contribute to future disaster response by participating in research.

To evaluate and develop the use of social media to conduct disaster health research, we suggest that the methodology needs to be further tested and used in other disaster contexts and timeframes as well as in follow up studies.

## Conclusions

Social media was a useful strategy to recruit participants for a self-selected Internet sample for disaster research and did limit several of the practical and ethical challenges connected to disaster research. However, the method demands access to Internet connection in the disaster area, several strategic considerations, and an active approach from the researcher. The method does not eliminate the problem with non-probability samples, which is common in disaster research. To fully understand the potential and use of social media for recruiting participants for disaster research, the strategy needs to be further used and evaluated.

## Competing Interests Statement

The authors have declared that no competing interests exist.

## Data Availability Statement

All available data has been reported in this article.

## Corresponding Author

Karin Hugelius, e-mail: karin.hugelius@oru.se
